# Flexible Asymmetric Supercapacitors Constructed by Reduced Graphene Oxide/MoO_3_ and MnO_2_ Electrochemically Deposited on Carbon Cloth

**DOI:** 10.3390/molecules29133116

**Published:** 2024-06-30

**Authors:** Sha Li, Zhiying Li

**Affiliations:** Department of Chemistry, Xinzhou Teachers University, Xinzhou 034000, China; lizhiying8001@163.com

**Keywords:** carbon cloth, reduced graphene oxide, MoO_3_, MnO_2_, asymmetric supercapacitors

## Abstract

A flexible asymmetric supercapacitor (ASC) is successfully developed by using the composite of MoO_3_ and graphene oxide (GO) electrochemically deposited on carbon cloth (CC) (MoO_3_/rGO/CC) as the cathode, the MnO_2_ deposited on CC (MnO_2_/CC) as the anode, and Na_2_SO_4_/polyvinyl alcohol (PVA) as the gel electrolyte. The results show that the introduction of the GO layer can remarkably increase the specific capacitance of MoO_3_ from 282.7 F g^−1^ to 341.0 F g^−1^. Furthermore, the combination of such good electrode materials and a neutral gel electrolyte renders the fabrication of high-performance ASC with a large operating potential difference of 1.6 V in a 0.5 mol L^−1^ Na_2_SO_4_ solution of water. Furthermore, the ASCs exhibit excellent cycle ability and the capacitance can maintain 87% of its initial value after 6000 cycles. The fact that a light-emitting diode can be lit up by the ASCs indicates the device’s potential applications as an energy storage device. The encouraging results demonstrate a promising application of the composite of MoO_3_ and GO in energy storage devices.

## 1. Introduction

Supercapacitors (SCs) are a potential energy storage device that have been widely studied due to their high energy density, fast charging and discharging speed, and long cycle life [1,2]. Supercapacitors are widely used in hybrid vehicles, backup energy systems, mobile electronic devices, and more [3]. With the increasing requirement for personal electronics in modern society, to make electronic devices flexible and portable has become the research hotspot at present. Correspondingly, flexible and portable electronic devices need design of a new type of energy storage device different from previous ones [4,5,6,7,8]. Therefore, flexible asymmetric SCs (ASCs) have attracted much attention, because they can provide more energy density compared with symmetric SCs [9,10,11,12,13,14,15].

To fabricate flexible ASCs (FASCs), it is essential and crucial to select the current collector and active materials because they directly affect the performance of FASCs [16,17,18]. As a new flexible conductor, carbon cloth (CC) has received much attention, due to its three-dimensional (3D) network structure and moderate electrochemical stability [19]. By using CC as a substrate, conducting polymers deposited on it has shown high energy density and power density. For example, Horng et al. have prepared polyaniline nanowire/CC nanocomposite electrodes by employing electrochemical deposition, and the PANI had a *C*_s_ of 1079 F g^−1^ [20]. An electrode of CC/MoS_2_/PANI has been prepared via combining the facile hydrothermal method, and the PANI of this electrode showed a *C*_s_ value of 972 F g^−1^ at 1 A g^−1^ [21].

Transition metal oxides are the other commonly used electrode materials for SCs; at first, the noble metal oxides such as RuO_2_ [22], RhO_x_ [23], etc., were extensively studied because of their high faradic capacitance and good conductivity. Despite the excellent capacitive performances, the rarity and high price of precious metal oxides limit their practicality. As a result, many studies have focused on the substitution of precious metal oxides due to their relative theoretical carbon content, availability of inexpensive transition metal oxides, and relative reversibility [24]. At present, layered porous manganese dioxide nanosheets with a maximum *C*_s_ of 268 F g^−1^ have been prepared by the rapid hydrothermal method [25]. Different from the usually used electrode material of MnO_2_, which possesses a capacitive property at a positive potential range, MoO_3_ exhibits capacitance at a negative potential range and has attracted much attention [26,27]. Furthermore, the other attractive nature of MoO_3_ is its particular 2D layered structure, which is conducive to provide high power density for capacitor applications because of the adequate intercalation between the layers and electrolyte ions [28]. Recently, Pujari et al. found that hexagonal MoO_3_ microrods display a *C*_s_ of 194 F g^−1^ [29,30]. Despite the efforts on MoO_3_, the *C*_s_ values are still not very high due to its low conductivity. Therefore, it is necessary to develop a strategy to prepare the composites with relatively high conductivity by adding some highly conductive materials [22,31,32,33,34]. The co-electrochemical deposition of active materials and carbon materials has been proven to be an effective method in various methods of manufacturing composite materials; as a precursor of graphene, graphene oxide (GO) has attracted great interest in the manufacturing of composite materials due to its ease of operation in aqueous media and high specific surface area [35,36].

In this work, we choose CC as a flexible substrate to deposit MoO_3_ as a cathode, during which the graphene oxide (GO) is added to form the composite of MoO_3_ and reduced GO (rGO) through electrochemical co-deposition. The electrode prepared by electrochemically depositing MnO_2_ on CC is used as an anode, MoO_3_/rGO/CC as a cathode, and NaSO_4_/polyvylene oxide as a gel electrolyte and separator to assemble ASCs. By using this strategy, we hope that the FASCs can possess high operating potential differences, energy density, and power density.

## 2. Results

### 2.1. Structural Properties’ Characterization of All Samples

**Figure 1:**
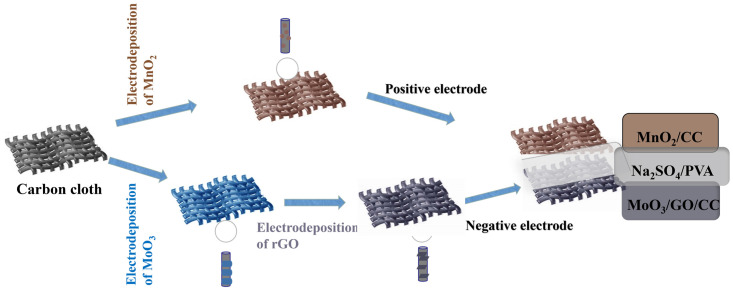
Schematic diagram for preparing MoO_3_/rGO/CC and MnO_2_/CC electrodes and assembled ASC.

Figure 1 depicts a schematic illustration of the procedure for the preparation of electrodes and the device. Based on the flexibility and conductivity of CC, and the potential ranges of electrochemical activity for MoO_3_ and MnO_2_ [37,38], the FASCs can be fabricated by using MoO_3_/rGO/CC as a cathode and MnO_2_/CC as an anode in a Na_2_SO_4_/PVA gel electrolyte.

**Figure 2:**
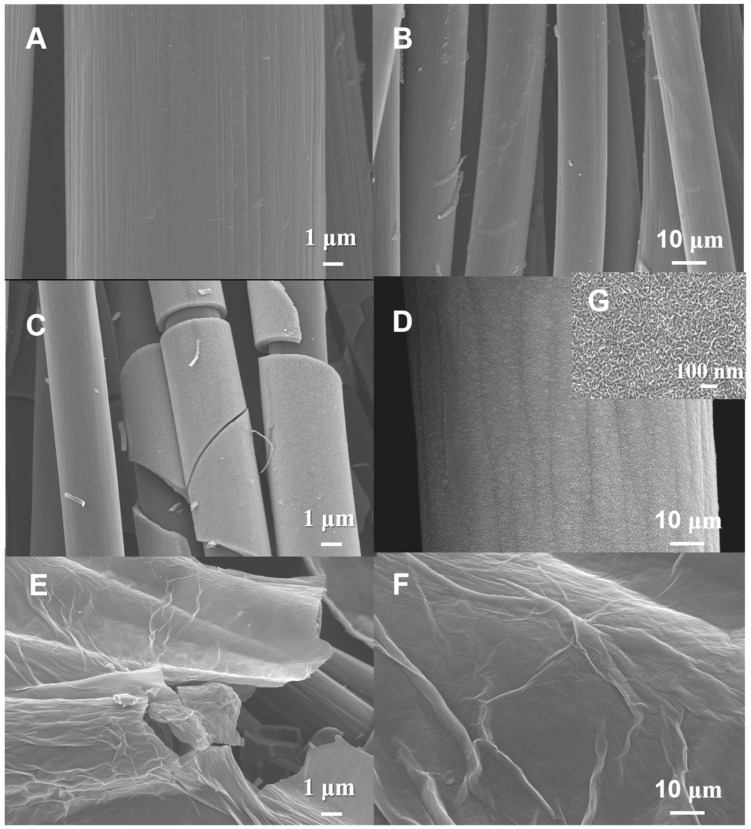
The SEM images in different magnification for CC (**A**,**B**), MoO_3_/CC (**C**,**D**), MoO_3_/rGO/CC (**E**,**F**), and inset of the enlarged image (**G**).

From the SEM image shown in Figure 2, it is found that fibers contained in CC have a clean surface with some groves (Figure 2A); the diameter of the fibers is relatively uniform and many accumulated pores are observed among the fibers (Figure 2B). When the molybdenum oxide has been electrochemically deposited, the surface of the fiber will be covered by a layer of molybdenum oxide; it can be seen from the image of MoO_3_/CC (Figure 2C) that the fiber is uniformly wrapped by a layer of molybdenum oxide, but the film has many cracks, which are caused during the drying process. Under the large magnification, a corrugated morphology is observed (Figure 2D); this rough surface structure is favorable to increase the surface area of MoO_3_. It is worth noting that by changing the film-forming time, the thickness and mass load of the film can be well controlled. When the evaporation time is 1000 s, a “skeleton/skin film” structure is obtained with carbon fiber as the skeleton and MoO_3_ as the skin film [39,40]. Figure S3 is the SEM image of the MnO_2_/CC electrode, from which it can be found that MnO_2_ particles are formed on the CC. In Figure 2E,F, the surface of CC is decorated with rGO flakes, embedded in the enlarged image (Figure 2G), which clearly shows that rGO smooths the surface of the carbon. In addition, uniform rGO nanosheets form a 2D layered structure on the surface of MoO_3_/rGO/CC, which increases the surface area and improves the electrical conductivity compared with the blank CC. SEM results showed that the surface of the MoO_3_/CC composite was successfully coated with a layer of rGO.

**Figure 3:**
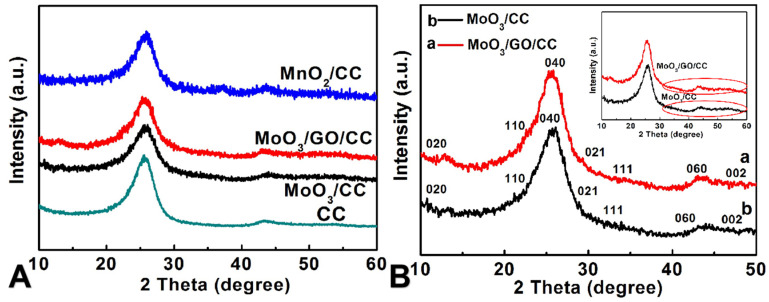
The XRD patterns of CC, MnO_2_/CC, MoO_3_/CC, and MoO_3_/rGO/CC (**A**); the magnified XRD patterns for MoO_3_/CC and MoO_3_/rGO/CC in the range of 10–50° (**B**).

To confirm the properties of the product, X-ray diffraction was used to evaluate the prepared material. From Figure 3, it can be found that all the samples show obvious peaks related to the substrate of CC or rGO, but no distinguishable peaks can be seen for the components of MoO_3_ and MnO_2_, which may be attributed to the fact that the formed MoO_3_ and MnO_2_ are amorphous or the crystalline is too small, or the weak diffraction peaks related to MoO_3_ and MnO_2_ are covered up by the diffraction peak of C due to the small amount of the active materials [19,41,42,43]. The FTIR spectrum provides details on the band characteristics of composite materials. As shown in Figure S4, no strong characteristic peaks are seen in the wave number ranging from 400 to 2000 cm^−1^. The spectrum of MoO_3_/CC shows three main peaks at 955, 826, and 753 cm^−1^. This is the Mo=O stretching vibration of terminal oxygen and the symmetric and asymmetric stretching vibration of Mo-O-Mo cross-linked oxygen, respectively [44]. However, the IR spectrum for GO/MoO_3_/CC exhibits the characteristic of rGO [19] besides the MoO_3_’s characteristics (C=C: 1738 cm^−1^), confirming that rGO has successfully loaded on the surface of MoO_3_/CC.

**Figure 4:**
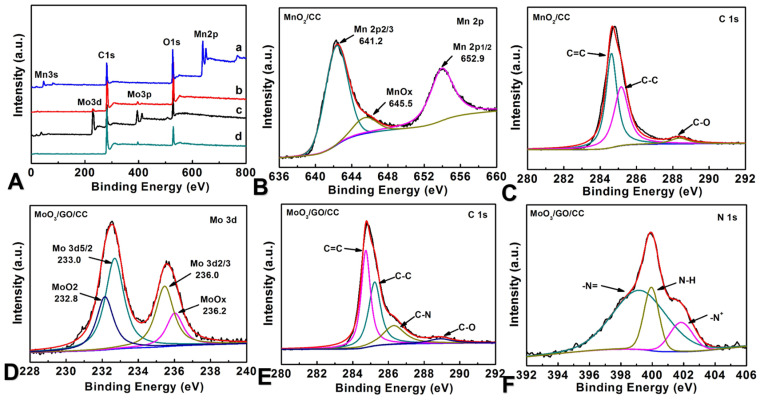
(**A**) The whole XPS spectra for MnO_2_/CC (a), MoO_3_/CC (b), MoO_3_/rGO/CC (c), and CC (d); (**B**) the high-resolution XPS for Mn2p in MnO_2_/CC; (**C**) the C1s XPS for MnO_2_/CC; (**D**) the high-resolution XPS for Mo3d in MoO_3_/rGO/CC; (**E**) the C1s XPS; and (**F**) N1s XPS for MoO_3_/rGO/CC.

The analysis of the XPS can reveal the chemical states of the samples of MnO_2_/CC (a), MoO_3_/rGO/CC (b), MoO_3_/CC (c), and CC (d) (Figure 4A). All the spectra show the XPS peaks related to the element of Mn or Mo together with that of C and O. Specially, the characteristic doublets of Mn2p (Figure 4B) 2p1/2 (654.1 eV) and Mn2p 2p3/2 (642.4 eV) from MnO_2_/CC identify the formation of MnO_2_ due to the difference between the two peaks of 11.7 eV [45]. Similarly, the double peaks observed at 233.0 and 236.2 eV in MoO_3_/rGO/CC can be contributed to Mo 3d5/2 and Mo 3d3/2, respectively, due to spin-orbit splitting (Figure 4D) [44]. According to the fitted results, it can be found that the components of MoO_2_ and MoO_x_ are exhibited in the sample besides the component of MoO_3_ [46]. On the other hand, the above peaks in the Mo 3d spectrum of MoO_3_/rGO/CC composite materials are shifted by 0.2 eV due to the interaction between rGO and MoO_3_, to 232.8 eV and 236.0 eV, respectively. The peak intensity of the MoO_3_/CC spectrum is higher than that of MoO_3_/rGO/CC.

### 2.2. The electrochemical Properties of MoO_3_/rGO/CC

The galvanostatic method is used to deposit MoO_3_ on the pretreated CC, and the depositing current and depositing time are optimized by changing the current and depositing time. Firstly, the deposition current is optimized in the same deposition solution by changing the current, keeping the depositing charge (Q = 6 C) amount constant. The obtained electrodes’ CV curves are tested by employing a three-electrode system, and according to the formulae shown in the Supplementary Materials, the plot of the *C*_m_ value versus the currents is drawn in Figure S1. The *C*_m_ value reaches its maximum value of 282.8 F g^−1^ at 6 mA. Subsequently, the current is fixed at 6 mA to change the deposition time to optimize the time. It can be found in Figure S1B that the maximal *C*_m_ value of 283.5 F g^−1^ is achieved when the depositing time is 1000 s. Therefore, we use the condition of a 6 mA constant current and depositing time of 1000 s to prepare the electrode in the following experiments. For improving the performance, electrodes of MoO_3_/CC are used as working electrodes to electrochemically deposit the rGO in a different GO solution; from Figure S1C, we can find that when the GO solution is 7.0 mg mL^−1^, the modified electrode shows the largest *C*_m_ value. Therefore, the electrode prepared at the above-mentioned condition will be evaluated in detail.

**Figure 5:**
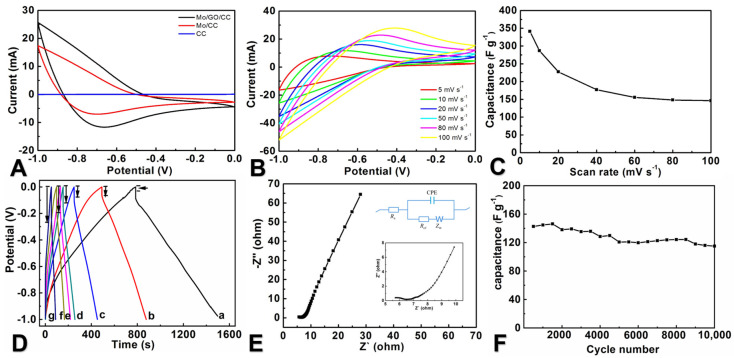
Electrochemical behaviors of the MoO_3_/rGO/CC electrode: (**A**) CV curves of CC, MoO_3_/CC, and MoO_3_/rGO/CC; (**B**) CV curves for the MoO_3_/rGO/CC electrode at different scan rates; (**C**) the plot of the C_m_ values versus scan rate for MoO_3_/rGO/CC; (**D**) GCD curves of the MoO_3_/rGO/CC electrode at different current densities (a–g represent GCD curves with current density of 1–7 mA cm^−2^, respectively); (**E**) EIS spectra for the MoO_3_/rGO/CC electrode (inset of E: equivalent circuit diagram of Nyquist plots); (**F**) the plot of capacitance versus the cyclic number for MoO_3_/rGO/CC. The electrolyte is a 0.5 mol L^−1^ Na_2_SO_4_ aqueous solution.

Figure 5A shows the electrode of MoO_3_/rGO/CC exhibiting far larger surrounded CV area than MoO_3_/CC at the same scan rate. The CV curve of the MoO_3_/rGO/CC electrode shows a similar shape within the range of a potential scanning rate of 5 to 100 mV s^−1^; based on the curves, the *C*_m_ values of MoO_3_/rGO/CC are calculated and shown in Figure 5C, and it is clear that the *C*_m_ values of MoO_3_/rGO/CC decrease with the increase in the scan rates because of the limited diffusion time for ions when the scan rate is large, and it has the largest *C*_m_ value of 341.0 F g^−1^ that is higher than that of MoO_3_/CC (282.8 F g^−1^) at 1 mV s^−1^, which is believed to be because the conductive rGO flakes on the surface of MoO_3_ shorten the transport pathways of electrons and electrolyte ions. As the scanning rate increases, the *C*_m_ value of MoO_3_/rGO/CC shows a decreasing trend. The GCD curves of the MoO_3_/rGO/CC electrode in a potential range of −1.0–0 V (Figure 5D) display almost symmetric shapes at different current densities; the *iR* drop is small at low current density and increases with the increment of the current densities, and the *C*_m_ obtained from the GCD curves are similar to those from CV profiles. The good capacitive performance of MoO_3_/rGO/CC can be due to the close contact between the carbon fiber and MoO_3_ layer, in addition to fast and effective charge transfer through a three-dimensional CC framework; graphene oxide sheets coated on MoO_3_ also provide an electron transfer pathway. At an open circuit potential, the EIS spectrum has been recorded within the frequency range from 100 kHz to 0.01 Hz to reveal the kinetic property of MoO_3_/rGO/CC.

Regarding the Nyquist curve (Figure 5E), the semicircles in the high-frequency region and the straight peaks in the low-frequency region are clearly seen [45,47,48]. The profile can be fitted by using Zview (2.3.1) software; the *R*_ct_ value of the GO/MoO_3_/CC electrode is 1.82 Ω, indicating the enhanced charge transportation provided by the decorated rGO on the surface. The electrochemical stability of the electrodes investigated by successive CV scanning at 100 mV s^−1^ (Figure 5F), and it is found that MoO_3_/rGO/CC can retain 88.6%, indicating better cycling stability.

### 2.3. Electrochemical Performance of the FASC

**Figure 6:**
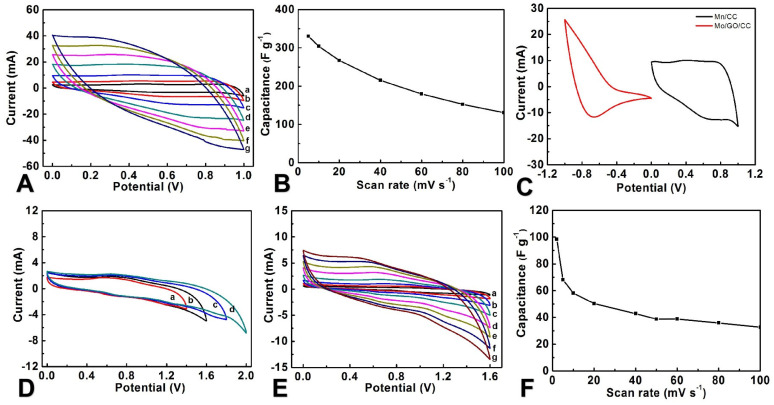
(**A**) CV curves for MnO_2_/CC at different scan rates (a-g represent scan rates of 5, 10, 20, 40, 60, 80, and 100 mV s^−1^, respectively), (**B**) the plot of C_m_ of MnO_2_/CC versus the scan rates, (**C**) the CV curves of the positive and negative electrodes in the three-electrode system at a scan rate of 20 mV s^−1^, (**D**) the FASC’s CV curves recorded at various potential difference windows at 20 mV s^−1^ (a-d represent 0–1.4, 1.6, 1.8, and 2.0 V, respectively), (**E**) the FASC’s CV curves recorded at different scan rates (a–g represent scan rates of 2, 5, 10, 20, 50, 80, and 100 mV s^−1^, respectively), (**F**) the plot of the FASC’s C_m_ based on active material versus scan rates.

The anode has been prepared by using CC as a substrate via the electrochemical depositing method. The conditions for depositing MnO_2_ have been optimized in Supplementary Materials (Figure S2). The electrode prepared at a current of 4 mA for 1000 s exhibits the optimal *C*_m_ value (343.3 F g^−1^), and the electrodes prepared under this condition are further investigated and used to assemble the devices. In a 0.5 mol L^−1^ Na_2_SO_4_ solution, the CV curve of the MnO_2_/CC electrode exhibits a quasi-rectangular shape at low scanning speeds, indicating excellent performance of MnO_2_/CC (Figure 6A). It can be easily seen from the curves of *C*_m_ and scanning speed that as the scanning speed increases, the C*_m_* value decreases. In 100 mV s^−1^, the C*_m_* value maintains 45.1% of the maximum C*_m_* value (Figure 6B), demonstrating good rate capability. Based on the results obtained from CV tests in the three-electrode system, the area ratio of MoO_3_/rGO/CC to MnO_2_/CC is 1:1. Therefore, the devices are constructed by using the Na_2_SO_4_/PVA gel electrolyte to separate the cathode of MoO_3_/rGO/CC and anode of MnO_2_/CC. By employing the two-electrode system, we recorded the CV curves in different voltage windows at 20 mV s^−1^ (Figure 6C,D) to determine the operating potential difference. It is clear that the operating potential difference of the device can be expanded to 1.6 V since MoO_3_/rGO/CC is −1.0–0 V, while MnO_2_/CC is 0–1.0 V, which are determined by the three-electrode system [49,50,51]. Notably, the CV curve of the ASC device is similar to the curve observed in the MnO_2_/CC, maintaining stable redox pairs within 2–100 mV s^−1^ (Figure 6E), and the scan rate increases and the C*_m_* of ASC decreases at 2 mV s^−1^ (Figure 6F); the device shows a C*_m_* of 98.3 F g^−1^ (C*_a_* of 226.4 mF cm^−2^).

**Figure 7:**
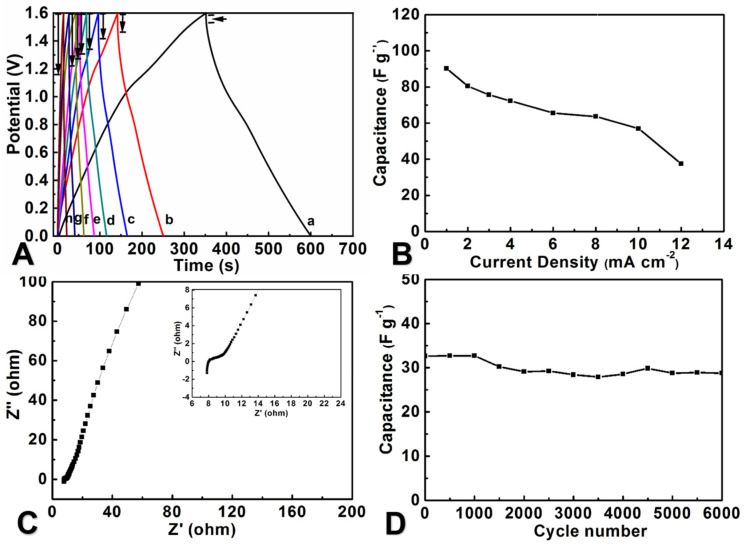
(**A**) GCD curves of ASC recorded at different current densities (a-h represent current densities of 1.0, 2.0, 3.0, 4.0, 6.0, 8.0, 10.0, and 12.0 mA cm^−2^), (**B**) the plot of specific capacitances versus current densities, (**C**) Nyquist plots for the ASC of MoO_3_/rGO/CC//MnO_2_/CC, (**D**) the plot of the capacitance of ASC versus the cyclic number.

The GCD curve (Figure 7A) shows a quasi-linear symmetric shape, with fast voltage current response and good electrochemical reversibility. Due to the presence of pseudo-capacitance in the device, the result deviates slightly from the straight line. At low current density, the GCD curve shows a tiny decrease in *iR*. At 1.0 mA cm^−2^, the device can achieve a C*_m_* of approximately 88.3 F g^−1^ and C*_a_* of 203.4 mF cm^−2^. The C*_m_* value of ASC decreases with the increase in current density (Figure 7B), which is analogous to the result of CV.

In order to further investigate the detailed electrochemical characteristics of ASC, we performed EIS experiments (Figure 7C). The result shows that at low frequencies, lines almost perpendicular to the Z′ axis exhibit quite good capacitance characteristics [52,53,54]. After 6000 cycles, the capacitance of the ASC device remained at 88.1% of the initial value and showed good cycling stability, indicating that this type of ASC device has good electrochemical stability (Figure 7D).

**Figure 8:**
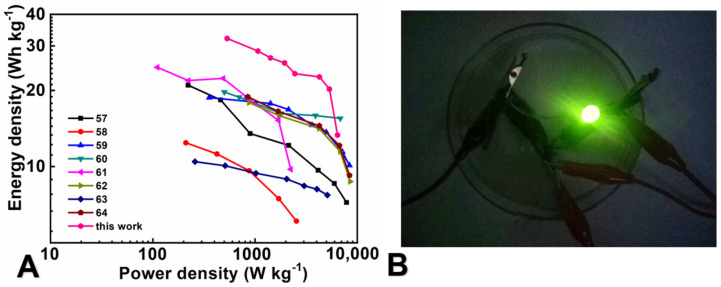
(**A**) The Ragone plots for ASC of MoO_3_/rGO/CC//MnO_2_/CC and some other devices from the previous literature for comparison [55,56,57,58,59,60,61,62]; (**B**) the photograph of an LED lit by two cells connected in series.

In order to evaluate the practical performance of the device, E and P were calculated based on the discharge branch of the GCD curve using the formula provided in the Supplementary Information (Figure 8A). At a P of 533.3 W kg^−1^, E is 32.1 Wh kg^−1^. More noteworthy is that when P reaches 5333.3 W kg^−1^, E remains at 20.2 Wh kg^−1^. Due to the high electrochemical capacitance and good multiplication ability of the electrode, it is superior to other devices previously reported (Table 1), indicating the potential application of this material. In addition, LEDs can be driven by fully charged series batteries (Figure 8B), indicating the possibility of practicality. By combining the pseudo-capacitive material fully loaded on the CC substrate and mild gel electrolyte, an effective way for preparing high-performance ASC with excellent cycling performance can be obtained.

## 3. Materials and Methods

### 3.1. Materials

Manganese (II) acetate (Mn(Ac)_2_·4H_2_O), ammonium molybdate [(NH_4_)_2_MoO_4_], ammonium chloride, polyvinyl alcohol (PVA, Mw: 85,000), and sodium sulfate (Na_2_SO_4_) were purchased from Aladdin Chemical Co. (Ontario, CA, USA) and used without further purification. The conductive carbon cloth (CC) substrate was obtained from Shanghai Chuxi Industrial Co., Ltd. (Shanghai, China)

### 3.2. Fabrication of the Electrodes of MnO_2_/CC and MoO_3_/rGO/CC

Pretreatment of the CC: CC is made from pre-oxidized polyacrylonitrile fabric that is carbonized or spun from carbon fiber. As a kind of flexible carbon-based template with special porous structure, high electrical conductivity, high mechanical stability, and corrosion resistance, carbon cloth not only has the inherent characteristics of carbon materials, but also has the machinability of fiber materials. It is often used as a substrate to carry active substances and improve the electrochemical performance of capacitors. The commercial CC was first immersed in concentrated nitric acid (HNO_3_, 68 wt.%) and then heated at 60 °C for about 6 h in a water bath to make the surface hydrophilic. Subsequently, the CC was washed with distilled water thoroughly and then soaked in distilled water for use.

Electrochemically depositing MnO_2_ on CC: By using a three-electrode system, MnO_2_/CC was also prepared via an electrodeposition method. In the mixing solution of 15 mL Mn(Ac)_2_ (0.05 mol L^−1^) and 15 mL Na_2_SO_4_ (0.05 mol L^−1^), the MnO_2_ was deposited on the pretreated CC at a constant current of 4.0 mA cm^−2^ when the CC was used as a working electrode, a Pt plate as a counter electrode, and a saturated calomel electrode (SCE) as a reference electrode, and the amount of MnO_2_ was controlled by depositing time (600–1400 s). After the deposition process was completed, the electrode of MnO_2_/CC was washed by deionized water and dried in a vacuum oven at 70 °C overnight. The electrode of MnO_2_/CC prepared from a different depositing time was defined as MnO_2_/CC-t (t is the depositing time).

Electrochemically depositing MoO_3_ on CC: The used electrolyte here (30.0 mL) was an aqueous solution of 15 mL (NH_4_)_2_MoO_4_ (0.05 mol L^−1^) and 15 mL NH_4_Cl (0.05 mol L^−1^) and its pH value was adjusted to 2.0 by acetic acid. The pretreated CC slices (1 × 1 cm^2^) were used as a working electrode, a Pt sheet as a counter electrode, and a saturated calomel electrode (SCE) as a reference electrode; by using a constant current density of 4 mA cm^−2^, different amounts of MoO_3_ were deposited on CC by controlling the depositing time (700–1300 s). According to the capacitance measured by using a three-electrode system in a 0.5 M Na_2_SO_4_ electrolyte, the MoO_3_/CC prepared from 1000 s was used to assemble the ASCs.

Electrochemically depositing MoO_3_/rGO on CC: First, GO was prepared by oxidizing 300-mesh graphite powder according to the modified hummer method [63]. The sample of MoO_3_/rGO/CC prepared by using the electrochemical deposition method according to the above was used as a work electrode here to deposit GO on the surface of MoO_3_/CC by the cyclic voltammetry (CV) method in different concentrations of the GO solution, and the obtained electrode was defined as MoO_3_/rGO/CC. The appropriate GO in the electrolyte is selected by optimizing the deposition time and the CV number. Place MoO_3_/rGO/CC in a vacuum oven overnight. The electrochemical performance test was carried out in a three-electrode system after drying.

### 3.3. Fabrication of the FASC Devices

Gel electrolyte: 1.0 g PVA powders were slowly added into a 10.0 mL solution of Na_2_SO_4_ (0.5 mol L^−1^) under stirring; then, the mixture was heated to 85 °C until the mixture became transparent, and then the solution was naturally cooled to room temperature for utilization.

Assembly of FASC device: Firstly, the CC was first pretreated with HNO_3_ at 60 °C to remove the sizing agent on the surface of the fibers. Then, MnO_2_/CC and MoO_3_/rGO/CC were fabricated using a facile electrodeposition method. MnO_2_ or MoO_3_ or rGO was grown on the CC substrate using three-electrode configuration with CC as the working electrode, a Pt piece as the counter electrode, and Hg/HgCl_2_ as the reference electrode. Finally, MoO_3_/rGO/CC was used as the negative electrode and MnO_2_/CC as the positive electrode. According to the capacitance measured by using the three-electrode system, it was clear that the capacitances of the MoO_3_/rGO/CC negative electrode and MnO_2_/CC positive electrode could be balanced when geometric area of the two electrodes was the same. When the FASC device was assembled, the Na_2_SO_4/_PVA gel electrolyte was uniformly covered on one electrode such as MnO_2_/CC, and then the other electrode (MoO_3_/rGO/CC) was placed on the face of MnO_2_/CC, which was covered by the Na_2_SO_4/_PVA gel electrolyte and followed by pressure to bring the two electrodes together; subsequently, the assembled devices were frozen in a refrigerator for 30 min. Finally, the fabrication of FASC was completed after the frozen device was placed at room temperature to unfreeze.

## 4. Conclusions

A convenient and efficient method for depositing high-active pseudo-capacitive materials on CC (MoO_3_/rGO/CC and MnO_2_/CC) has been developed. The introduction of the rGO layer can remarkably improve the specific capacitance of the MoO_3_ layer on the carbon fibers from 282.7 to 341.0 F g^−1^. Furthermore, the assembled ASC by using the electrochemically deposited electrodes and neutral gel electrolyte Na_2_SO_4_/PVA possesses a large operation potential of 1.6 V, and exhibits a high energy density of 32.08 Wh kg^−1^ at the power density of 0.53 kW kg^−1^, and 5.33 Wh kg^−1^ at 20.2 kW kg^−1^. Furthermore, the ASC exhibits good cycle ability and the capacitance can maintain 87.1% of its initial value after 6000 cycles. The ability of these two ASC devices connected in series is that they are able to light up an LED, which indicates their potential applications as energy storage devices.

## Figures and Tables

**Table 1:** Comparison of the energy density and cyclic stability of the ASCs.

Electrode Materials	Electrolyte	Energy Density (Wh kg^−1^)	Stability (Capacitance Retention %, Cycles)	Ref.
MoO_3_-PPy/CNTs-MnO_2_	Na_2_SO_4_/PVA gel	21.0	76.0, 10,000 cycles	[55]
MnO_2_@PEDOT/PEDOT	LiClO_4_/PMMA gel	9.8	86.0, 1250 cycles	[56]
Fe_3_O_4_ embedded in carbon nanosheet/porous carbon	KOH/PVA gel	18.3	70.8, 5000 cycles	[57]
Graphene (IL-CMG)/RuO_2_-IL-CMG	H_2_SO_4_/PVA gel	19.7	95.0, 2000 cycles	[58]
CNTs/MnO_2_/CNTs/PANI	Na_2_SO_4_/PVP gel	24.8	-	[59]
Co_3_O_4_ nanowires/Ni foam/ carbon aerogel	KOH/PVA gel	17.9	-	[60]
Mn_3_O_4_ nanoparticle/graphene/CNT/graphene	KCl/PVA gel	32.7	86.0, 10,000 cycles	[61]
Graphene/Ni(OH)_2_/graphene/CNT	KOH/PVA gel	18	-	[62]
MoO_3_/rGO/CC/MnO_2_/CC	Na_2_SO_4_/PVA gel	32.1	88.1, 6000 cycles	This work

## Data Availability

The data presented in this study are available either in this article or in the Supplementary Materials.

## Supplementary Materials

The following supporting information can be downloaded at: https://www.mdpi.com/article/10.3390/molecules29133116/s1, Figure S1. Specific capacitance variations of MoO_3_/CC versus deposition currents, time, and concentrations of GO. Figure S2. The plots of specific capacitance of MnO_2_/CC versus depositing currents and times. Figure S3. The SEM images in different magnification for MnO_2_/CC (A,B). Figure S4. The FTIR spectra of MoO_3_/CC and MoO_3_/rGO/CC.

## References

[1] He W., Wu B., Lu M., Li Z., Qiang H. (2020). Fabrication and Performance of Self-Supported Flexible Cellulose Nanofibrils/Reduced Graphene Oxide Supercapacitor Electrode Materials. Molecules.

[2] Zhou G., Liang G., Xiao W., Tian L., Zhang Y., Hu R., Wang Y. (2024). Porous α-Fe_2_O_3_ Hollow Rods/Reduced Graphene Oxide Composites Templated by MoO_3_ Nanobelts for High-Performance Supercapacitor Applications. Molecules.

[3] Saha S., Samanta P., Murmu N.C., Kuila T. (2018). A review on the heterostructure nanomaterials for supercapacitor application. J. Energy Storage.

[4] Liu Z., Xu J., Chen D., Shen G. (2015). Flexible electronics based on inorganic nanowires. Chem. Soc. Rev..

[5] Kim B.C., Hong J., Wallace G.G., Park H.S. (2015). Recent Progress in Flexible Electrochemical Capacitors: Electrode Materials, Device Configuration, and Functions. Adv. Energy Mater..

[6] Wang X., Lu X., Liu B., Chen D., Tong Y., Shen G. (2014). Flexible Energy-Storage Devices: Design Consideration and Recent Progress. Adv. Mater..

[7] Lu X., Yu M., Wang G., Tong Y., Li Y. (2014). Flexible solid-state supercapacitors: Design, fabrication and applications. Energy Environ. Sci..

[8] Li L., Wu Z., Yuan S., Zhang X.-B. (2014). Advances and challenges for flexible energy storage and conversion devices and systems. Energy Environ. Sci..

[9] He W.D., Wang C.G., Li H.Q., Deng X.L., Xu X.J., Zhai T.Y. (2017). Ultrathin and Porous Ni_3_S_2_/CoNi_2_S_4_ 3D-Network Structure for Superhigh Energy Density Asymmetric Supercapacitors. Adv. Energy Mater..

[10] Guan C., Zhao W., Hu Y., Lai Z., Li X., Sun S., Zhang H., Cheetham A.K., Wang J. (2017). Cobalt oxide and N-doped carbon nanosheets derived from a single two-dimensional metal–organic framework precursor and their application in flexible asymmetric supercapacitors. Nanoscale Horiz..

[11] Jabeen N., Hussain A., Xia Q.Y., Sun S., Zhu J.W., Xia H. (2017). High-Performance 2.6 V Aqueous Asymmetric Supercapacitors based on In Situ Formed Na_0.5_MnO_2_ Nanosheet Assembled Nanowall Arrays. Adv. Mater..

[12] El-Kady M.F., Kaner R.B. (2013). Scalable fabrication of high-power graphene micro-supercapacitors for flexible and on-chip energy storage. Nat. Commun..

[13] Gwon H., Kim H.-S., Lee K.U., Seo D.-H., Park Y.C., Lee Y.-S., Ahn B.T., Kang K. (2011). Flexible energy storage devices based on graphene paper. Energy Environ. Sci..

[14] Lee S.W., Kim B.-S., Chen S., Shao-Horn Y., Hammond P.T. (2009). Layer-by-Layer Assembly of All Carbon Nanotube Ultrathin Films for Electrochemical Applications. J. Am. Chem. Soc..

[15] Zhu Y., Murali S., Stoller M.D., Ganesh K.J., Cai W., Ferreira P.J., Pirkle A., Wallace R.M., Cychosz K.A., Thommes M. (2011). Carbon-Based Supercapacitors Produced by Activation of Graphene. Science.

[16] Staaf L., Lundgren P., Enoksson P. (2014). Present and future supercapacitor carbon electrode materials for improved energy storage used in intelligent wireless sensor systems. Nano Energy.

[17] Huang Y., Huang Y., Zhu M.S., Meng W.J., Pei Z.X., Liu C., Hu H., Zhi C.Y. (2015). Magnetic-Assisted, Self-Healable, Yarn-Based Supercapacitor. ACS Nano.

[18] Wang M., Yan Q., Xue F., Zhang J., Wang J. (2018). Design and synthesis of carbon nanotubes/carbon fiber/reduced graphene oxide/MnO_2_ flexible electrode material for supercapacitors. J. Phys. Chem. Solids.

[19] Ling J., Zou H., Yang W., Chen W., Lei K., Chen S. (2018). Facile fabrication of polyaniline/molybdenum trioxide/activated carbon cloth composite for supercapacitors. J. Energy Storage.

[20] Horng Y.-Y., Lu Y.-C., Hsu Y.-K., Chen C.-C., Chen L.-C., Chen K.-H. (2010). Flexible supercapacitor based on polyaniline nanowires/carbon cloth with both high gravimetric and area-normalized capacitance. J. Power Sources.

[21] Zhang H., Qin G., Lin Y., Zhang D., Liao H., Li Z., Tian J., Wu Q. (2018). A novel flexible electrode with coaxial sandwich structure based polyaniline-coated MoS_2_ nanoflakes on activated carbon cloth. Electrochim. Acta.

[22] Mendoza-Sánchez B., Brousse T., Ramirez-Castro C., Nicolosi V., Grant P.S. (2013). An investigation of nanostructured thin film α-MoO_3_ based supercapacitor electrodes in an aqueous electrolyte. Electrochim. Acta.

[23] Liu T., Pell W., Conway B. (1997). Self-discharge and potential recovery phenomena at thermally and electrochemically prepared RuO_2_ supercapacitor electrodes. Electrochim. Acta.

[24] Klink M., Makgae M., Crouch A. (2010). Physico-chemical and electrochemical characterization of Ti/RhO_x_–IrO_2_ electrodes using sol–gel technology. Mater. Chem. Phys..

[25] Zhang X., Yu P., Zhang H., Zhang D., Sun X., Ma Y. (2013). Rapid hydrothermal synthesis of hierarchical nanostructures assembled from ultrathin birnessite-type MnO_2_ nanosheets for supercapacitor applications. Electrochim. Acta.

[26] Cao X., Zheng B., Shi W., Yang J., Fan Z., Luo Z., Rui X., Chen B., Yan Q., Zhang H. (2015). Reduced graphene oxide-wrapped MoO_3_ composites prepared by using metal–organic frameworks as precursor for all-solid-state flexible supercapacitors. Adv. Mater..

[27] Barzegar F., Bello A., Momodu D.Y., Dangbegnon J.K., Taghizadeh F., Madito M.J., Masikhwa T.M., Manyala N. (2015). Asymmetric supercapacitor based on an α-MoO_3_ cathode and porous activated carbon anode materials. RSC Adv..

[28] Zhang Y., Lin B., Wang J., Han P., Xu T., Sun Y., Zhang X., Yang H. (2016). Polyoxometalates@Metal-Organic Frameworks Derived Porous MoO_3_@CuO as Electrodes for Symmetric All-Solid-State Supercapacitor. Electrochim. Acta.

[29] Li J., Liu X. (2013). Preparation and characterization of α-MoO_3_ nanobelt and its application in supercapacitor. Mater. Lett..

[30] Pujari R.B., Lokhande V.C., Kumbhar V.S., Chodankar N.R., Lokhande C.D. (2015). Hexagonal microrods architectured MoO_3_ thin film for supercapacitor application. J. Mater. Sci. Mater. Electron..

[31] Liang R., Cao H., Qian D. (2011). MoO_3_ nanowires as electrochemical pseudocapacitor materials. Chem. Commun..

[32] Li G.-R., Wang Z.-L., Zheng F.-L., Ou Y.-N., Tong Y.-X. (2011). ZnO@MoO_3_ core/shell nanocables: Facile electrochemical synthesis and enhanced supercapacitor performances. J. Mater. Chem..

[33] Wang S., Dou K., Dong Y., Zou Y., Zeng H. (2016). Supercapacitor based on few-layer MoO_3_ nanosheets prepared by solvothermal method. Int. J. Nanomanuf..

[34] Icaza J.C., Guduru R.K. (2017). Characterization of α-MoO_3_ anode with aqueous beryllium sulfate for supercapacitors. J. Alloys Compd..

[35] Huang M., Li F., Dong F., Zhang Y.X., Zhang L.L. (2015). MnO_2_-based nanostructures for high-performance supercapacitors. J. Mater. Chem. A.

[36] Yao W., Wang J., Li H. (2014). Flexible α-MnO_2_ paper formed by millimeter-long nanowires for supercapacitor electrodes. J. Power Sources.

[37] Chang J., Jin M., Yao F., Kim T.H., Le V.T., Yue H., Gunes F., Li B., Ghosh A., Xie S. (2013). Asymmetric Supercapacitors Based on Graphene/MnO_2_ Nanospheres and Graphene/MoO_3_ Nanosheets with High Energy Density. Adv. Funct. Mater..

[38] Greiner M.T., Helander M.G., Tang W.-M., Wang Z.-B., Qiu J., Lu Z.-H. (2011). Universal energy-level alignment of molecules on metal oxides. Nat. Mater..

[39] Zhang Z., Chi K., Xiao F., Wang S. (2015). Advanced solid-state asymmetric supercapacitors based on 3D graphene/MnO_2_ and graphene/polypyrrole hybrid architectures. J. Mater. Chem. A.

[40] Lu K., Song B., Li K., Zhang J., Ma H. (2017). Cobalt hexacyanoferrate nanoparticles and MoO_3_ thin films grown on carbon fiber cloth for efficient flexible hybrid supercapacitor. J. Power Sources.

[41] Han S., Lin L., Zhang K., Luo L., Peng X., Hu N. (2017). ZnWO_4_ nanoflakes decorated NiCo_2_O_4_ nanoneedle arrays grown on carbon cloth as supercapacitor electrodes. Mater. Lett..

[42] Li Z., Ding Y., Xiong Y., Yang Q., Xie Y. (2005). One-step solution-based catalytic route to fabricate novel α-MnO_2_hierarchical structures on a large scale. Chem. Commun..

[43] Noh J., Yoon C.-M., Kim Y.K., Jang J. (2017). High performance asymmetric supercapacitor twisted from carbon fiber/MnO_2_ and carbon fiber/MoO_3_. Carbon.

[44] Jiang F., Li W., Zou R., Liu Q., Xu K., An L., Hu J. (2014). MoO_3_/PANI coaxial heterostructure nanobelts by in situ polymerization for high performance supercapacitors. Nano Energy.

[45] Choi B.G., Yang M., Hong W.H., Choi J.W., Huh Y.S. (2012). 3D macroporous graphene frameworks for supercapacitors with high Energy and Power Densities. ACS Nano.

[46] Zhou K., Zhou W., Liu X., Sang Y., Ji S., Li W., Lu J., Li L., Niu W., Liu H. (2015). Ultrathin MoO_3_ nanocrystalsself-assembled on graphene nanosheets via oxygen bonding as supercapacitor electrodes of high capacitance and long cycle life. Nano Energy.

[47] Cao H., Wu N., Liu Y., Wang S., Du W., Liu J. (2017). Facile synthesis of rod-like manganese molybdate crystallines with two-dimentional nanoflakes for supercapacitor application. Electrochim. Acta.

[48] Senthilkumar B., Selvan R.K. (2014). Hydrothermal synthesis and electrochemical performances of 1.7V NiMoO_4_⋅xH_2_O||FeMoO_4_ aqueous hybrid supercapacitor. J. Colloid Interface Sci..

[49] Lv Q.Y., Wang S., Sun H. (2016). Solid-State Thin-Film Supercapacitors with Ultrafast Charge/Discharge Based on N-Doped-Carbon-Tubes/Au-Nanoparticles-Doped-MnO_2_ Nanocomposites. Nano Lett..

[50] Cheng J., Sprik M. (2012). Alignment of electronic energy levels at electrochemical interfaces. Phys. Chem. Chem. Phys..

[51] Lee J.S., Shin D.H., Jang J. (2015). Polypyrrole-coated manganese dioxide with multiscale architectures for ultrahigh capacity energy storage. Energy Environ. Sci..

[52] Lee J.S., Shin D.H., Jun J., Lee C., Jang J. (2014). Fe_3_O_4_/Carbon Hybrid Nanoparticle Electrodes for High-Capacity Electrochemical Capacitors. ChemSusChem.

[53] Kim S.-K., Kim Y.K., Lee H., Lee S.B., Park H.S. (2014). Back Cover: Superior Pseudocapacitive Behavior of Confined Lignin Nanocrystals for Renewable Energy-Storage Materials (ChemSusChem 4/2014). ChemSusChem.

[54] Li S., Chang Y., Han G., Song H., Chang Y., Xiao Y. (2019). Asymmetric supercapacitor based on reduced graphene oxide/MnO_2_ and polypyrrole deposited on carbon foam derived from melamine sponge. J. Phys. Chem. Solids.

[55] Du P., Wei W., Liu D., Kang H., Liu C., Liu P. (2018). Fabrication of hierarchical MoO_3_–PPy core–shell nanobelts and “worm-like” MWNTs–MnO_2_ core–shell materials for high-performance asymmetric supercapacitor. J. Mater. Sci..

[56] Duay J., Gillette E., Liu R., Lee S.B. (2012). Highly flexible pseudocapacitor based on freestanding heterogeneous MnO_2_/conductive polymer nanowire arrays. Phys. Chem. Chem. Phys..

[57] Fan H., Niu R., Duan J., Liu W., Shen W. (2016). Fe_3_O_4_@Carbon nanosheets for all-solid-state supercapacitor electrodes. ACS Appl. Mater. Interfaces.

[58] Choi B.G., Chang S.-J., Kang H.-W., Park C.P., Kim H.J., Hong W.H., Lee S., Huh Y.S. (2012). High performance of a solid-state flexible asymmetric supercapacitor based on graphene films. Nanoscale.

[59] Jin Y., Chen H., Chen M., Liu N., Li Q. (2013). Graphene-patched CNT/MnO_2_ nanocomposite papers for the electrode of high-performance flexible asymmetric supercapacitors. ACS Appl. Mater. Interfaces.

[60] Liu W., Li X., Zhu M., He X. (2015). High-performance all-solid state asymmetric supercapacitor based on Co_3_O_4_ nanowires and carbon aerogel. J. Power Sources.

[61] Gao H., Xiao F., Ching C.B., Duan H. (2012). Flexible all-solid-state asymmetric supercapacitors based on free-standing carbon nanotube/graphene and Mn_3_O_4_nanoparticle/graphene paper electrodes. ACS Appl. Mater. Interfaces.

[62] Li M., Tang Z., Leng M., Xue J. (2014). Flexible solid-state supercapacitor based on graphene-based hybrid films. Adv. Funct. Mater..

[63] Xie Y., Cheng Z., Guo B., Qiu Y., Fan H., Sun S., Wu T., Jin L., Fan L. (2015). Preparation of activated carbon paper by modified Hummer’s method and application as vanadium redox battery. Ionics.

