# Supplementary material for: Flexible Asymmetric Supercapacitors Constructed by Reduced Graphene Oxide/MoO3 and MnO2 Electrochemically Deposited on Carbon Cloth
Source: Molecules. 2024 Jun 30;29(13):3116. doi: 10.3390/molecules29133116 (PMC11243340; doi:10.3390/molecules29133116)
Supplement: Supplementary file 1 [file molecules-29-03116-s001.zip › molecules-3047933-supplementary.pdf]

Supporting information for the following:

Flexible Asymmetric Supercapacitors Constructed by Reduced Graphene Oxide/MoO3 and MnO2 Electrochemically Deposited on Carbon Cloth

Sha Li and Zhiying Li

Department of Chemistry, Xinzhou Teachers University, Xinzhou 034000, China

1. Characterization and electrochemical measurements

The electrodes' morphologies were observed by using a scanning electron microscope (SEM, JEOL 6701) operating at an accelerating voltage of 10 kV. Attenuated total reflection Fourier transforming infrared spectra (ATR-FTIR) were collected on a Nicolet iS50R (USA) instrument in a range of 400-4000 cm-1 with a resolution of 2 cm^{-1}. X-ray photoelectron spectroscopy (XPS ESCAL-ab 220i-XL, VG Scientific, England) was performed by using monochromic Al Ka as a source.

The capacitive measurements of the electrode materials and the ASCs were carried out on a CHI 660E electrochemical work station. A three-electrode system was used to test the electrodes' capacitive performance; the prepared positive and negative electrodes were used as the working electrode, a Pt sheet as the counter electrode, and an SCE as the reference electrode. An aqueous solution of Na_2SO_4 (0.5 M) was used as an electrolyte for testing MnO_2 and MoO_3 capacitive properties, respectively. The potential windows were set to 0-1.0 V for the positive electrode (MnO_2) and -1.0-0 V for the negative electrode (MoO_3) during the cyclic voltammetry (CV) and galvanostatic charge/discharge (GCD) tests. Using the two-electrode system, the performances of the FASCs were evaluated. The CV measurements were carried out in a voltage range of 0.0-1.6 V and the scan rates were varied from 2 to 100 mV s^{-1}. The GCD tests were performed at various current densities from 1.0 to 12.0 mA cm^{-2} with a cut-off voltage of 0.0-1.6 V. In the frequency range of 0.01-100000 Hz, the electrochemical impedance spectra (EIS) were measured by employing a disturbance with a 5 mV potential amplitude. The cyclic stabilities were estimated on the same instrument by a successive CV scan at 100 mV s^{-1}.

2. computational formulae

The specific capacitance in geometric area (C_a) and in mass (C_m) for the electrodes tested by employing the three-electrode system could be calculated based on formula (S1) and (S2):

$$C_a = \frac{\int IdV}{2 \cdot v \cdot S \cdot \Delta V} \quad \text{(S1)}$$

$$C_m = \frac{\int IdV}{2 \cdot v \cdot m \cdot \Delta V} \quad \text{(S2)}$$

where C_a is in terms of mF cm^{-2}, and C_m in F g^{-1}; $\int IdV$ is the integrated areas surrounded by CV curves, S is the geometric area of the electrode calculated by length × width (cm^2), ΔV is the voltage difference during the CV tests (V), v is the potential scan rate (V s^{-1}), and m is the loading mass of the active materials (g).

Moreover, the C_a and C_m of the single electrode could also be obtained from the GCD curves using formula (S3) and (S4):

$$C_a = \frac{I \cdot t}{S \cdot \Delta V} \quad \text{(S3)}$$

$$C_m = \frac{I \cdot t}{m \cdot \Delta V} \quad \text{(S4)}$$

where I is the constant discharge current (A), t is the discharging time (s), and other parameters are the same as formula (S1) and (S2).

In order to evaluate the performances of the ASCs, the C_m for the cells tested by the two-electrode system could be calculated based on formulae (S5) and (S6):

$$C_m = \frac{\int IdV}{2 \cdot v \cdot m \cdot \Delta V} \quad \text{(S5)}$$

where m is the active substance mass of the positive and negative electrodes in the asymmetric capacitor (g), and other parameters are the same as formula (S1) and (S2).

The C_a could also be obtained from the GCD curves using formula (S6):

$$C_m = \frac{I \cdot t}{m \cdot \Delta V} \quad \text{(S6)}$$

To further determine the properties of the materials, the energy density and power density [51] of the ASCs depicted in the Ragone plot were required, which could be calculated by using formula (S7) and (S8) according to the ASC's GCD curves, respectively.

$$E = \frac{1}{2} \cdot \frac{C_m \Delta V^2}{3600} \quad \text{(S7)}$$

$$P = \frac{3600E}{t} \quad \text{(S8)}$$

where E is the mass energy density in Wh kg^{-1}, P is the mass power density in W kg^{-1}, C_m is the specific capacitance in F g^{-1}, V is the voltage window (excluding the iR drop at the beginning of the discharge) in V, and t is the discharge time in s.

The Q using formula (S9) is as follows:

$$Q = It \quad \text{(S9)}$$

where Q is the charge (C), I is the constant discharge current (A), and t is the discharging time (s).

To prepare the ASC device, the capacitance of the negative electrode must match

that of the positive electrode. The matching of the two electrodes' capacitance can be determined by the charge balance relationship of $Q_+ = Q_-$, where Q_+ is the charge stored in the positive electrode and Q- is the charge in the negative electrode. To the flexible devices, the charge storage at each electrode relies on the C_a, operating voltage window (ΔV), and geometric surface area (S); the charge storage can be given by equation (10):

$$Q = C_a \times \Delta V \times S \quad \text{(S10)}$$

To satisfy $Q_+ = Q_-$, the areal balance can be expressed by using the relationship represented as (11):

$$\frac{S_+}{S_-} = \frac{C_- \times \Delta V_-}{C_+ \times \Delta V_+} \quad \text{(S11)}$$

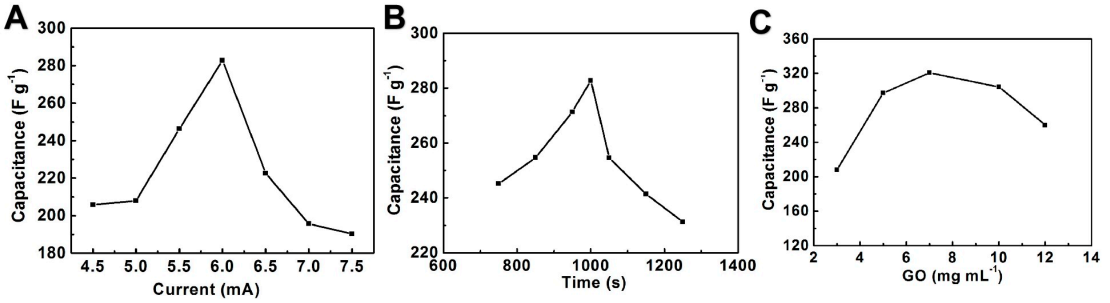

Figure S1. Specific capacitance variations of MoO_3/CC versus deposition currents, time, and concentrations of GO.

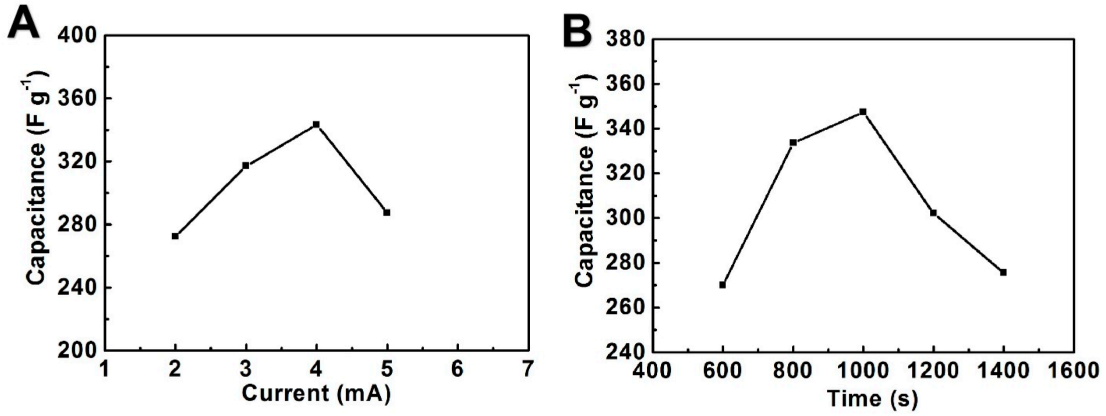

Figure S2. The plots of specific capacitance of MnO_2/CC versus depositing currents and times.

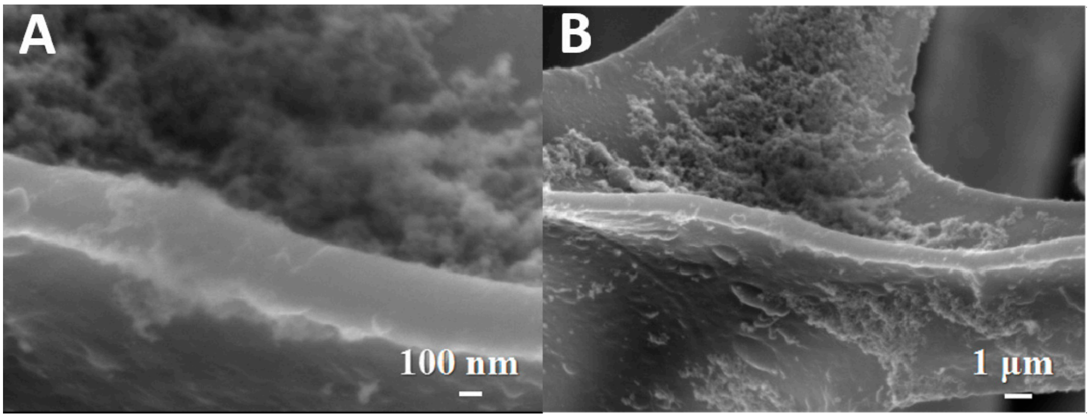

Figure S3. The SEM images in different magnification for MnO_2/CC (A, B).

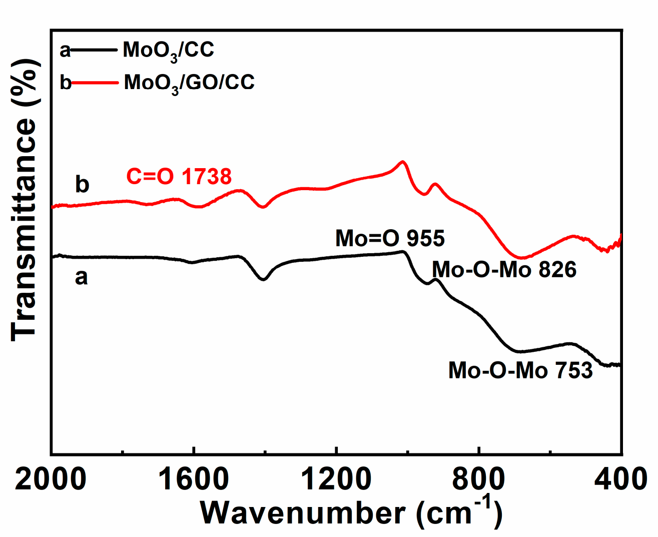

Figure S4. The FTIR spectra of MoO_3/CC and MoO_3/rGO/CC.
